# ARIA: insights from human neuropathology

**DOI:** 10.1002/alz70861_108867

**Published:** 2025-12-23

**Authors:** Delphine Boche

**Affiliations:** ^1^ University of Southampton, Southampton, Hampshire UK

## Abstract

**Background:**

Side effects of Aβ immunotherapy (ARIA) have hampered initial trials and subsequent clinical use. The pathophysiology is poorly understood because of lack of human tissue samples. AN1792 was the first anti‐Aβ immunotherapy, relying on the patient's own immune system to generate anti‐Aβ antibodies using the full‐length Aβ peptide. Although different from current passive immunotherapy agents, both approaches aim to produce plaque‐binding anti‐Aβ antibodies, leading to side effects that appear similar.

**Method:**

Postmortem neuropathological studies of 22 patients with mild to moderate Alzheimer's disease (AD) enrolled in the first clinical trial of Aβ immunotherapy (AN1792).

**Result:**

Potentially beneficial changes in most treated patients included plaque clearance, microglial phagocytosis of Aβ, reduced tau pathology, straightening of neuronal processes and reduced dystrophic neurites. However, continued spread of tau putatively explained continuing cognitive decline. Vascular changes included increased severity of cerebral amyloid angiopathy, but also removal of vascular Aβ and microhemorrhages. One of the 22 patients had features now recognised as ARIA including cortical microhemorrhages (ARIA‐H), focal white matter changes (ARIA‐E) and CAA‐related inflammation (CAA‐ri).

**Conclusion:**

Evidence suggests that vascular factors underlie the pathophysiology of ARIA, particularly immunological attack directed at CAA. The few available case reports of side effects of currently licenced passive immunotherapy support these conclusions. Study of spontaneous CAA‐ri may help better understand ARIA.

